# Research and Remedies

**Published:** 1912-10-26

**Authors:** 


### RESEARCH AND REMEDIES.

### The Sputum Diagnosis of Pneumonia.

A simple test for pneumonia, which is useful in the early stages before the typical rusty tinge is developed, is described by Pacini in the *Interstate Medical Journal*. A portion of sputum is placed in ten times its volume of distilled water, agitated, and filtered. Then take a test-tube containing 10 c.c. of distilled water, add five drops of a 1-per-cent. aqueous solution of methyl violet, and mix thoroughly. Then add drop by drop ten minims of the filtrate from the sputum; if a distinct red colour is produced the reaction is positive. The author states that the reaction is due to a specific disintegrated blood pigment present in the sputum. He has applied the test to 1,200 cases of pneumonia, and finds that it can be relied upon in 98 per cent. of the cases. He is still engaged upon further investigations into the chemistry and physiology of this simple and easily applied reaction.

